# Serious Games and Gamification for Mental Health: Current Status and Promising Directions

**DOI:** 10.3389/fpsyt.2016.00215

**Published:** 2017-01-10

**Authors:** Theresa M. Fleming, Lynda Bavin, Karolina Stasiak, Eve Hermansson-Webb, Sally N. Merry, Colleen Cheek, Mathijs Lucassen, Ho Ming Lau, Britta Pollmuller, Sarah Hetrick

**Affiliations:** ^1^Department of Psychological Medicine, University of Auckland, Auckland, New Zealand; ^2^Department of Paediatrics: Child and Youth Health, University of Auckland, Auckland, New Zealand; ^3^University of Tasmania Rural Clinical School, Burnie, TAS, Australia; ^4^Department of Health, Wellbeing and Social Care, The Open University, Milton Keynes, UK; ^5^Department of Psychiatry, VU University Medical Center, Amsterdam, Netherlands; ^6^School of Art and Design, Auckland University of Technology, Auckland, New Zealand; ^7^Orygen Youth Health Research Centre, Centre for Youth Mental Health, University of Melbourne, Parkville, VIC, Australia

**Keywords:** serious gaming, games for health, gamification, computerized CBT, e-therapy, engagement

## Abstract

Computer games are ubiquitous and can be utilized for serious purposes such as health and education. “Applied games” including serious games (in brief, computerized games for serious purposes) and gamification (gaming elements used outside of games) have the potential to increase the impact of mental health internet interventions *via* three processes. First, by extending the reach of online programs to those who might not otherwise use them. Second, by improving engagement through both game-based and “serious” motivational dynamics. Third, by utilizing varied mechanisms for change, including therapeutic processes and gaming features. In this scoping review, we aim to advance the field by exploring the potential and opportunities available in this area. We review engagement factors which may be exploited and demonstrate that there is promising evidence of effectiveness for serious games for depression from contemporary systematic reviews. We illustrate six major categories of tested applied games for mental health (exergames, virtual reality, cognitive behavior therapy-based games, entertainment games, biofeedback, and cognitive training games) and demonstrate that it is feasible to translate traditional evidence-based interventions into computer gaming formats and to exploit features of computer games for therapeutic change. Applied games have considerable potential for increasing the impact of online interventions for mental health. However, there are few independent trials, and direct comparisons of game-based and non-game-based interventions are lacking. Further research, faster iterations, rapid testing, non-traditional collaborations, and user-centered approaches are needed to respond to diverse user needs and preferences in rapidly changing environments.

## Introduction

Computer games are played by millions of adolescents (1) and adults (2) around the world, with over 40% of the United States population playing computer games for 3 or more hours per week in 2015 (2). Computer games vary enormously along dimensions such goals, interaction, and involved technologies. They include fast mini-games, as simple as lining up dots in a row, through to augmented reality (AR), and intricate shared worlds. Quality computer games have been shown to enhance concentration (3), improve retention of information (4), facilitate deep learning (5), and bring about behavior change (6). Over recent decades, computerized game-based approaches, both “serious games” and “gamification” have been developed for “serious” purposes: to educate, motivate, and/or persuade users, in educational, health, and other settings (7, 8). “Serious games” and “gamification” can be defined variously. However, both seek to employ games (or substantial game elements) in an effort to educate and change patterns of experience and/or behavior. Serious games utilize gaming as a central and primary medium (9). In contrast, gamification refers to the addition of game elements to non-game contexts (10). A gamified intervention may not operate as a full game experience but contains gaming elements, such as the scoring of points, in-game rewards, or engaging in quests.

Game-based approaches for mental health are in their infancy. However, initial studies, mainly of serious games, suggest potential benefits for psychological and behavioral changes, or symptom relief (11–17). Alongside these scientific developments, there has been significant growth in smartphone apps for mental health (18–20). Some of these use gaming or gamification, but most have not been scientifically tested (18).

To date, the potential of serious games and gamification (together “applied games” or “applied gaming”) in mental health has been understudied. Where applied games have been researched, interventions are often poorly described, and diverse approaches are treated as homogeneous. In this perspective paper, we aim to advance the field by highlighting the scope of applied games for mental health. We consider the potential for applied gaming and motivational features that may be utilized. We examine evidence of effectiveness from systematic reviews and demonstrate major types of tested applied games for mental health. Finally, we highlight promising directions for development.

## The Potential of Applied Games

Applied games are intriguing for mental health for three reasons:

First, applied gaming offers “*appealing potential*,” as suggested by the popularity of computer games (21). Applied gaming approaches might increase the reach of mental health interventions to some who might not otherwise access help. This is important given the large numbers of people who experience mental distress and yet receive no treatment (the mental health treatment gap) (22).
Second, applied gaming has “*engaging potential*.” Users might experience gaming approaches as enjoyable, want to “win” the game, or see how the story unfolds. Such dynamics may contribute to reducing high attrition rates in naturalistically implemented internet-based interventions (23, 24).
Third, applied gaming has “*effectiveness potential*,” because it provides opportunities for both conventional and non-traditional processes for behavior change and learning. For instance, applied gaming can offer immersive experiences where a state of “flow” can be achieved, provide rich sensory environments to support learning, allow behavioral modeling and social learning, allow users to try new skills in a safe yet reactive environment, and facilitate repeated rehearsal of new behavior (9, 25–27).

## Engagement

Games exploit varied processes for engagement. Hamari and Tuunanen (28) carried out a meta-synthesis of 12 studies and identified key motivational orientations that support engagement. These were achievement, exploration, sociability, domination, and immersion (for examples, see Table 2). A further motivation of escape (where a user plays to escape real-life problems) has also been reported (29). A user may have several or all of these motivations for playing a game, and predominant motivations may vary across demographic groups, contexts, and types of game (28). This model could be extended to reflect the proposition that users of applied games might also have a further dimension along which their motivation varies, that of the serious purpose itself, in the present case, interest in improving mental health. For users who are motivated in this way, it may be that just a few gaming elements might enhance engagement or even that gaming elements are off-putting (30). For those less motivated to improve their mental health, stronger and more extensive gaming features may be critical.

## Existing Research: A Scoping Review

In order to provide an overview of evidence for applied games, we carried out a review of systematic reviews. Our inclusion criteria were systematic review of serious games and/or gamified interventions for mental health (treatment or prevention), published in the peer-reviewed literature from 2010 to June 2016 (reflecting the recent development of the field), and available in English. We searched PsycINFO and Medline using the terms (systematic review) AND (mental health OR mental illness OR depression OR anxiety) AND (treatment OR prevention) AND (computer OR internet OR digital OR online) AND (game OR gaming OR gamification OR play). After duplicates were removed, a total of 18 papers were identified. A Google search and check of citations yielded no further papers for inclusion. Titles and abstracts were independently scanned by two authors (TF and LB). From these, three systematic reviews, two of which also included meta-analyses, fitted the inclusion criteria (see Table 1).

**Table 1:** **Systematic reviews of applied games for mental health**.

Reference	Scope	Inclusion criteria	Included programs (brief description)	Conclusions
Li et al. (31)	Systematic review and meta-analysis of exergames for depression	Studies of exergames to treat or prevent depression. Search terms and detailed inclusion criteria specified. Inclusion criteria include published in peer-reviewed literature and reliable and valid measures of depression symptoms at pre- and post-intervention. Non-RCTs included	Nine studies, eight using Wii Sports, Wii Fit or Wii Fit Plus, or other commercial exergames and one purpose-built rehabilitation game. Each tested for impact on depression or depressive symptoms among at-risk groups (mainly among elderly)	Small, but significant effect of exergames on depressive symptoms. Larger scale robust studies needed

Fleming et al. (9)	Systematic review of serious games for the treatment or prevention of depression	Studies of computer games/serious games to treat or prevent depression. Search terms and detailed inclusion criteria specified. Inclusion criteria include published in peer-reviewed literature and reliable and valid measures of depression symptoms at pre- and post-intervention. Non-RCTs included	Nine studies related to six programs: Think Feel Do: 6 module computerized cognitive behavior therapy (cCBT) program aimed at children/adolescents with depression or emotional distress, delivered on a personal computer (PC), includes game-like elements SPARX: 7 module cCBT program for adolescents with depression. Delivered on a PC. Utilizes a virtual therapist and fantasy world setting with overarching narrative and play-based learning. Rainbow SPARX: modified version of SPARX for sexual minority youth with depression The Journey: 7 module cCBT program for adolescents with depression. Delivered on a PC. Utilizes 2D fantasy setting, mini-games, and puzzles with overarching narrative gNAT island: cognitive behavior therapy (CBT)-based program delivered over 2–4 sessions with a therapist Journey to the Wild Divine: a “Freeze-Framer” game-based biofeedback in fantasy setting ReachOutCentral: a non-modular program involving interpersonal problem solving and role-playing, with the story of being new in town. Utilizes principles of CBT	Most studies reported promising results, although one universal program (ReachOutCentral) had mixed results. Interventions show promise, but the evidence is currently very limited

Li et al. (32)	Systematic review and meta-analysis of game-based digital interventions for depression	Studies of game-based digital applications for depression. Search terms not specified and inclusion criteria are not detailed; however, they must include reliable and valid measures of depression symptoms. Surveys, non-RCTs, and single case studies were included	Identified 19 studies including: Exercise games for depression among older adults, using Nintendo Wii [as included in Ref. (31)] SPARX, ReachOutCentral, and Journey to the Wild Divine [as included in Ref. (9)] Virtual reality interventions for PTSD, phobia, or depression Entertainment games tested for impact on psychological symptoms Programs that are not described as games or gamified by the original authors such as Beating the Blues	Findings support effectiveness, but further research is needed

Each of the included papers was focused specifically on depression. Two reviews (9, 32) examined serious games for depression, while Li et al. (31) focused specifically on a subset of serious games, exergames. Fleming et al. (9) identified nine studies of six computerized interventions that utilized gaming as a major or primary component to treat or prevent depression. Li et al. (32) identified a higher number of studies, although exactly how many studies of how many interventions was not clearly reported and the search terms and inclusion criteria were not specified. As shown in Table 1, both Li et al. (32) and Fleming et al. (9) conclude that the utilization of serious games for depression is promising, but that further research is needed. Given the heterogeneity of included studies and the nature of many of these being small trials, some not randomized or controlled, stronger conclusions would be premature. In the more narrowly focused review (31), only three Randomised Controlled Trials (RCTs) were identified; however, the authors reported a significant effect of exergames on depressive symptoms. As noted in each review, there was a lack of direct comparison of game-based to non-gaming interventions, and most studies were not independent of the developers. The research is at an early stage.

## Types of Applied Games

The three systematic reviews included six main types of applied games. Each of these categories is outlined below. An example of each, and potential mechanisms for therapeutic change and engagement, is given in Table 2.

**Table 2:** **Examples of major types of tested applied games for mental health**.

Types of game	Example	Main therapeutic modality	Increasing engagement					
			Examples of game-focused engagement features (facilitating engagement *via* user game-related motivations)					Serious purpose engagement features
			Achievement	Exploration	Sociability	Domination	Immersion	
Exergames	Nintendo Wii Sports (33)	Exercise, perhaps behavioral activation, social activity	Improve performance on sports games to increase the avatar’s skill level and to turn “pro.” Features fitness test	Explore different virtual sports settings	Can play with others	Compete against others to win tournaments	Requires real-time movement to play the game. Real-time performance feedback	Not described

Virtual reality	Virtual Iraq (34)	Exposure therapy	Habituate to progressively more provocative elements to progress through the recreated virtual environment	User navigates through virtual 3D simulation of combat environments	N/A	Confront provocative elements in the traumatic scenario to gain control over emotional responses	Immersive sensory 3D experience (rich 3D graphics and audio, olfactory, and vibrotactile stimuli)	Clinician provides rationale

Cognitive behavior therapy (CBT)-based serious games	SPARX (16)	CBT	Complete quizzes, shoot gNats (gloomy negative automatic thoughts), and find gems to ultimately restore balance to the virtual world	Explore virtual world	Player interacts with virtual guide/therapist and other in-game characters	Defeat gNats	Interactive narrative (“a hero to save the world”). Rich graphics	Virtual guide explains how the game is helpful for difficulties and can be applied in real-life

CBT-based gamification	SuperBetter (35)	CBT and positive psychology	Gain points and “level-up.” Complete quests and power-ups. Defeat “bad guys”	N/A	Facebook integration and online forums. Encourages connections with allies	Defeat “bad guys” (by overcoming specific obstacles)	Fun bite-sized activities. Can create own power-ups and quests	Program explicitly provides rationale for why intervention helps with resilience and mood

Biofeedback	Journey to the Wild Divine (36)	Psychoeducation and relaxation-based exercises paired with biofeedback	See progress over time *via* high-score tracking. Control physiology in order to successfully perform virtual activities, such as building a bridge or shooting a bow and arrow	Explore serene virtual worlds/environments	Encounter various guides and mentors in the virtual environment	N/A	Controlling physiology to play the game. Rich graphics and immersive sound	In-game explanation about how program works to prevent or relieve stress and enhance well-being

Entertainment computer games for mental health	Tetris (37)	Redirection of cognitive resources	Clear lines to successfully level-up	N/A	Can play against others and watch tournaments. Online forums	Defeat other players in multi-level modes and competitions. Leaderboards	Playing against the clock (time pressure)	Therapist may provide explanation

### Exergames

Exergames are sport or movement-based games. Nine of the included studies (across the three reviews) tested the use of exergames for depressive symptoms, primarily among older adults (31). Eight studies used “repurposed games,” games developed for entertainment or commercial purposes and now tested for mental health. One was purpose-built. Li et al. (31) reported a significant effect of exergames on depressive symptoms, with this being higher among more playful games, over those that included less game elements. However, these results should be interpreted with caution, given that only three of the studies were RCTs, and the sample sizes were small.

### Virtual Reality Games

Virtual Reality (VR) and Augmented Reality (AR) can offer immersive interactivity in a virtual or augmented world, with visual, audio, and sometimes other sensory stimuli, to increase user engagement and possibly therapeutic impact (38, 39). Li et al. (32) identified six studies of VR gaming interventions. They reported that these had positive results. However, only 2 had over 10 participants, and most of the original papers provide little detail regarding the game. Hence an alternative example, that of Virtual Iraq for PTSD (34) is given in Table 2. Promising findings from VR interventions, including non-game-based VR interventions (40) along with the popularity of commercial AR games, suggest promise in this area.

### Cognitive Behavior Therapy (CBT)-Based Serious Games and Gamification

Five of the interventions identified in the systematic reviews were multilevel CBT-based programs, often utilizing a fantasy environment, and designed to be completed at a rate of approximately one level per week on a personal computer. Each of these programs was aimed at children or young people. Each reported positive or promising results, except for ReachOutCentral, which had mixed findings and has since been retired. Of these, SPARX is described in the greatest depth and is outlined in Table 2.

A further example, SuperBetter, has been tested since the publication of the systematic reviews; however, it is included here (Table 2), as it illustrates new opportunities for applied games. SuperBetter is a positive psychology program in which players earn points and “level-up,” as they progress through activities. Rather than being a narrative-based serious game, SuperBetter offers a more gamified approach, with scoring and rewards. In another point of difference, SuperBetter allows “snacktivity,” frequent, brief activities that can be done a few minutes at a time, every day or more often (therefore like “snacking” behavior). This pattern of use is common in contemporary online apps but is not common in tested online mental health tools to date, many of which follow traditional clinical therapeutic models (e.g., weekly sessions of 30 minutes or more). In a recent RCT, participants who were asked to play SuperBetter for 10 minutes daily over 30 days experienced significantly greater reductions in depressive symptoms and anxiety compared to a waitlist control group; however, attrition was high (35).

### Entertainment Computer Games

This category of interventions is quite different from those that translate an evidence-based mental health therapy (such as CBT or exposure therapy) into a game format. In this grouping, entertainment video games were tested for effects on mood. Several studies of this nature were included in Li et al. (32). In the first of these, students were given a “frustrating task” and then 45 minutes of violent video game play or a control condition. Those playing the violent game reported lower symptoms of depression immediately following the intervention in one study (41). However, in a second study, no effect for depression was reported (42). In a further study, Rossoniello et al. (43) reported subjects had improved mood immediately following playing a casual video game Bejeweled II. These entertainment games were proposed to affect mood *via* emotional regulation, stress release, or social support pathways.

A different form of using commercial games for mental health is the use of the puzzle game Tetris for therapeutic purposes. In Tetris, players strategically move, rotate, and drop “Tertriminos” to complete horizontal lines. Engaging in this visuospatial cognitive activity when memories are activated is proposed to help impede traumatic flashbacks in PTSD by interfering with memory consolidation (37). Preliminary findings have also shown promise for using Tetris to reduce cravings (44).

### Biofeedback-Based Games

The included reviews described two biofeedback-based games: the Journey to the Wild Divine and Freeze-Framer 2.0. In each of these, users rehearse relaxation skills while receiving visual feedback on physiological indicators (measured using a sensor attached to the ear lobe or the fingertips). In a small trial, youth receiving the intervention had significantly lower post-intervention levels of depression and anxiety compared to those in a waitlist control group (36).

### Cognitive Training Games

The reviews included one study using cognitive training games. Alvarez et al. (45) tested number and letter sequence training games to reduce cognitive impairment in 31 depressed students. In this study, the game had positive results on cognitive impairment, but direct effects on mood were not tested.

## Future Directions

It is demonstrably feasible to translate traditional evidence-based interventions, such as CBT and exposure therapies, to computer gaming formats. Included interventions have shown that it is also possible to exploit features of computer games for therapeutic change using mechanisms that are not traditionally salient in psychological therapies, such as in the example of Tetris for PTSD. Further, the potential for positive mental health outcomes from casual play of entertainment games is worthy of exploration, as this might offer opportunities to reach large numbers of people. Each of these approaches appears to be promising. However, this evidence is at an early stage, and independent, larger robust studies are needed. Further, there is a lack of data regarding whether gaming-based approaches might be more appealing than non-gaming mental health interventions for users with different motivations, including both those who do and those who do not want to access help for distress. This is a question for future research. Similarly, findings from trials and user reviews of some commercially available programs suggest that applied gaming approaches can be engaging; however, many analyses do not report engagement or ongoing use. The assertion that quality gaming dynamics will increase engagement, at least for some users, should also be tested in future research.

We have highlighted that applied gaming interventions vary widely, in terms of types of games and in terms of features that might be appealing and motivating. It would be valuable to explore popular engaging game types for target groups and compare features in those games with those used in games for mental health. For example, highly accessed games currently include smart phone-based mini-games, massive multiplayer games where millions of players interact, games that allow user-generated content, and games that are linked to popular social media platforms (2, 46, 47). These approaches did not feature strongly in the included interventions.

Despite the potential for applied gaming, there are challenges in proposing such approaches, including costs, speed of implementation, issues of face validity, and user preferences. Many gamers will be familiar with commercially produced games, which often involve development budgets in the tens of millions of dollars (48, 49). Even simple mini-games undergo rapid advances, with new versions regularly released. Funding limitations make it challenging to develop and maintain comparable scientific initiatives.

Speed of implementation is also critical (49). Traditional scientific methods of sequential development, piloting, refinement, testing in a RCT, followed by publication, and independent replication, prior to real-world implementation, will ensure that evidence-based applied gaming lags behind the rapidly shifting commercial hardware and software environments. Newer methodological approaches are available (24).

Rather different challenges include those of face validity and diverse user motivations. People often do not seek help for mental health issues until these are relatively serious (22), in which case a game may be perceived as trivializing or inappropriate (Fleming et al., manuscript in preparation). Conversely, those who do not want therapeutic help might be irked that their game has a mental health agenda as suggested by the gamergate phenomena (50). These factors should be investigated in future research. The diversity of gaming approaches and user motivations also pose challenges. Not all games are successful. Quality game development requires specialist skills, and meeting user preferences may necessitate the creation of a range of interventions.

We have previously proposed four key ways of maximizing the impact of E-therapies and serious games in mental health. Utilizing this framework, as identified by the Collaboration on Maximizing the impact of E-therapy and Serious Gaming (COMETS) (24), we believe that to achieve significant mental health benefits, the field requires:

– User-centered approaches. This necessitates exploring the motivations and preferences of user groups for addressing their mental health needs. The field of game development has illustrated that this is unlikely to be through one approach or single engagement factor for all users.
– Engaging, as well as effective interventions. Even very effective interventions will have limited population impact if they are not engaging. Studies should explore and report engagement as well as effectiveness and provide sufficient detail regarding dynamics used, to allow others to build on their work.
– Intersectorial and international collaborations. The skills required to develop engaging and effective games with high uptake are diverse and go beyond many science or clinically focused teams. Further, the costs of developing interventions may be more easily borne across sectors and jurisdictions.
– Rapid testing and implementation. User expectations in technology-driven approaches and gaming evolve rapidly. Innovative, rapid research designs and planning for implementation are needed to ensure that interventions are still appealing when they are ready for implementation.

## Conclusion

In this perspective paper, we have illustrated the potential of serious games and gamification for mental health and highlighted that there is serious work still to be done. The field is ready for further development, as the feasibility and range of possible approaches has been shown, and as there is an urgent need for engaging, appealing effective mental health interventions which reach large numbers of people. Future research should include independent trials and direct comparisons of game-based and non-game-based options for varied user groups.

## Author Contributions

TF drafted the paper, coordinated the input from other authors, and was responsible for the full submission. LB assisted with drafting and completing all aspects of the paper. All other authors contributed substantially to the draft and approved final submission.

## Conflict of Interest Statement

The intellectual property for SPARX is held by UniServices at the University of Auckland. Any proceeds from licensing or selling SPARX outside of New Zealand will be shared in part with UniServices and SM, KS, TF, M. Shepherd, and ML.
